# Diode Laser and Polyimide Tape Enables Cheap and Fast Fabrication of Flexible Microfluidic Sensing Devices

**DOI:** 10.3390/mi13122214

**Published:** 2022-12-14

**Authors:** Thana Thaweeskulchai, Albert Schulte

**Affiliations:** School of Biomolecular Science and Engineering, Vidyasirimedhi Institute of Science and Technology (VISTEC), Wang Chan Valley, Rayong 21210, Thailand

**Keywords:** diode laser, polyimide, CNC, PDMS, microfluidic, electrochemical analysis, wearable device, laser-induced graphene, electrode, flexible

## Abstract

Wearable devices are a new class of healthcare monitoring devices designed for use in close contact with the patient’s body. Such devices must be flexible to follow the contours of human anatomy. With numerous potential applications, a wide variety of flexible wearable devices have been created, taking various forms and functions. Therefore, different fabrication techniques and materials are employed, resulting in fragmentation of the list of equipment and materials needed to make different devices. This study attempted to simplify and streamline the fabrication process of all key components, including microfluidic chip and flexible electrode units. A combination of diode laser CNC machine and polyimide tape is used to fabricate flexible microfluidic chip and laser-induced graphene (LIG) electrodes, to create flexible microfluidic sensing devices. Laser ablation on polyimide tape can directly create microfluidic features on either PDMS substrates or LIG electrodes. The two components can be assembled to form a flexible microfluidic sensing device that can perform basic electrochemical analysis and conform to curved surfaces while undergoing microfluidic flow. This study has shown that simple, commonly available equipment and materials can be used to fabricate flexible microfluidic sensing devices quickly and easily, which is highly suitable for rapid prototyping of wearable devices.

## 1. Introduction

The advent of personalized medicine has pushed the boundaries of the healthcare industry, creating ripple effects in how healthcare is being delivered to patients. At the most personal level is the use of a new class of medical diagnostic tools called wearable devices, which are applied directly to specific parts of the body to provide continuous, noninvasive healthcare monitoring. The benefits of using wearable devices for healthcare monitoring include real-time feedback and comprehensive data sets that carry a whole range of information on biochemical dynamics of targeted biomarkers in test subjects or patients undergoing different conditions [1].

Wearable devices come in a variety of shapes and forms, depending on their functions and application sites on the body. In turn, the fabrication techniques and materials involved can be equally varied. However, they share some common physical structures and traits necessary for the devices to conform to and maintain close contact with the body for optimal collection and analysis of body fluids and corresponding biomarkers of interest. Mainly, they are flexible, stretchable or shaped to fit to the body. For example, a ring-shaped wearable device has been made with 3D printing to monitor glucose levels in sweat [2]. In other studies [3,4,5], a flexible PDMS microfluidic platform with screen-printed sensing elements was used to sample and analyze sweat. Other forms of wearable devices include contact lenses for analysis of glucose in tears [6] and a graphene-based dental patch sensor for detecting bacteria [7].

In several notable reviews [8,9], laser scribing has been shown to effectively produce graphene layers on polyimide material for a range of applications. The mechanism of graphene production involves either photothermal or photochemical processes, depending on the type of laser used [10,11,12,13]. In general, laser ablation on polyimide causes *sp^3^* carbon structures to transform into a *sp^2^* carbon lattice, as confirmed by Raman spectroscopy [14,15]. Results from high-resolution X-ray photoelectron spectroscopy (XPS) indicated that the laser ablation process breaks down C-C, C=O and N-C bonds in the polyimide structure, with nitrogen and oxygen components released as gas while aromatic carbon compounds are rearranged into graphene layers [16]. A readily available polyimide material is Kapton tape, which can be readily found in electronic and microfabrication facilities, where the prototyping and testing of microfluidic devices are performed.

In recent years, there has been increased interest in laser-induced graphene (LIG) and its application in wearable and flexible microfluidics. Examples of some research on the use of LIG are electrochemical detection of glucose [17], sound sensing in an artificial throat [12], and antibacterial membranes for industrial water filtration [18], as well as a wide range of medical diagnostic applications [19,20,21], including analysis of sweat samples [22,23,24]. Unmodified LIG has been shown to have the potential to perform basic electrochemistry. Interdigitated laser-induced graphene has been designed to function as microsupercapacitors where graphene layers serve as both active electrode and current collectors, showing double-layer capacitive activities [16]. A high-resolution laser has also been used to create LIG for the detection of humidity in human breath with a microsecond response time [11]. Modification of LIG working electrodes is possible and can vastly expand the scope of target detection. Examples of LIG modifications are Prussian blue [23] and platinum sputtering [25] for hydrogen peroxide detection, platinum nanoparticles [24] and platinum–silver nanoparticles with glucose oxidase on a flexible substrate [20] for glucose detection in sweat and crosslinking of poly(vinyl alcohol) for detection of bacteria [18].With diverse and varied forms and functions of flexible microfluidic devices, the fabrication protocols are also widely different from one another, depending on the design specifications, resources and equipment available to the investigator.

With real-world use, cases and applications, particularly in the biomedical sector, the process of combining flexible microfluidic device with LIG on flexible substrate to produce functional flexible microfluidic sensing units can offer new opportunities for the development of new point-of-care (POC) diagnostic devices. This work attempts to streamline and simplify the fabrication process by using a minimal range of materials and equipment that is affordable and easily accessible to fabricate both microfluidic components and sensing electrodes for the creation of flexible microfluidic electrochemical detection units. The aim is to promote rapid prototyping of flexible microfluidics by limiting both the start-up cost and the operating cost per unit. This protocol should allow increased adoption and participation in microfluidic research by removing barriers to entry that would previously have prevented the involvement of interested parties.

## 2. Materials and Methods

### 2.1. CAD Designs and Laser Fabrication Procedure

Autodesk Fusion 360 (Mill Valley, CA, USA) CAD software was used in sketching microfluidic designs and LIGs to be fabricated using the Snapmaker 2.0 Modular 3-in-1 3D Printer A250 with laser module producing 1600 mW of power at 450 nm wavelength (Shenzhen, China). Two-dimensional sketches of microfluidic designs and LIGs were created and exported in dxf file format. The files were then imported into Snapmaker Luban software (version 4.3.0), where laser settings for microfluidic and LIG fabrications can be controlled. The Fill method laser setting was used to control the laser module performing ablation within the specified area to create LIG and microfluidic features. Additional settings include Movement Mode set to Line and Fill Interval set at 0.05 mm. Laser height was set to 24 mm by default with auto-focus setting selected by manually inputting the combined thickness of substrate with a layer of Kapton film tape applied on top. Safety goggles were worn for the duration of laser operations.

### 2.2. LIG Fabrication

**Figure 1:**
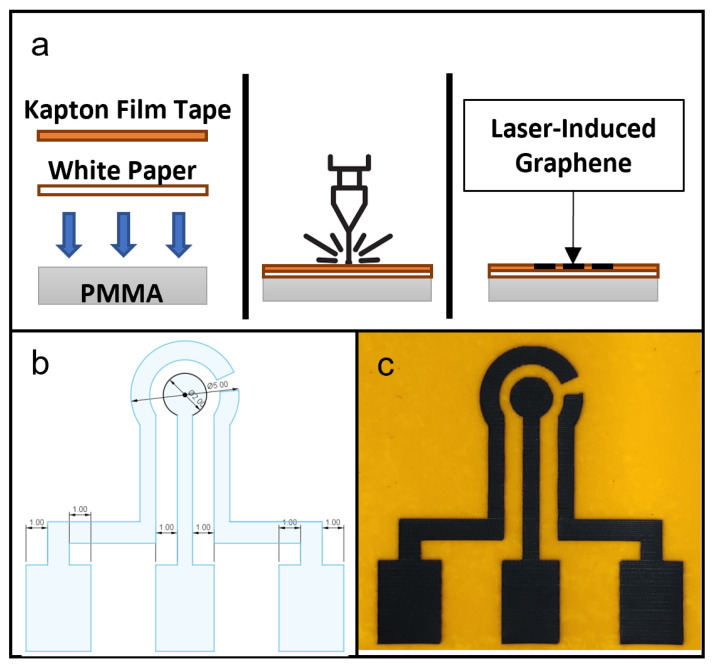
(**a**) Schematic of fabrication of laser-induced graphene (LIG) showing in cross-section the setup of materials, laser ablation process and finished product. (**b**) CAD drawing of a LIG electrode design for this study. (**c**) Photograph of a produced LIG electrode unit, with counter electrode (CE) on the left, working electrode (WE) in the center and reference electrode (RE) on the right.

A PMMA sheet, 1 mm thick, was used during fabrication by laser ablation as a flat mounting surface for flexible substrate layers adherent to Kapton film tape, to create an even, flat surface for consistent laser focusing and ablation. The polyimide material used in this study was 3M™ Polyimide Film Tape 5413 (Saint Paul, MN, USA), and the flexible substrate layer was a piece of white paper, cut to size. The PMMA sheet was placed at the bottom, with a piece of printing paper above it and the Kapton film tape used as the adhesive to both hold the paper onto the PMMA sheet and also provide the top surface material for laser-induced graphene production. The fabrication scheme is shown in Figure 1a.

The design of LIG used in this study is shown in Figure 1b. The protocol for LIG fabrication was based on a study by Tao et al. [12]. In Snapmaker Luban software, the laser setting used was the Fill method, with work speed set at 500 mm/min and laser powers set at 100, 150, 200, 250 and 300 mW. Manual focusing was performed and the laser height was set to 24 mm.

### 2.3. Electrochemical Characterization

The potentiostat used in this study was a Palmsens 4 (Houten, The Netherlands). Electrochemical tests were performed with analyte in bulk solution, dropped directly onto the LIG electrodes. The tests performed were cyclic voltammetry (CV) and differential pulse voltammetry (DPV) with various concentrations of potassium ferricyanide solution and different test parameters. Later, CV was performed in microfluidic channels under static conditions with the same analyte solution for comparison.

### 2.4. LIG Characterization

A confocal Raman microscope, Bruker RamanScope Senterra (Billerica, MA, USA), with bundled software (Opus version 7.5.18) was used to examine the LIG surface to verify formation of graphene structures on polyimide film. The region to be analyzed was on the central circular area to be used as working electrode. Settings used were laser wavelength 532 nm, laser power 20 mW, circular aperture of diameter 50 µm, resolution 3–5 cm^−1^, and 5 co-additions with exposure time 15 s [10].

### 2.5. Laser Settings for Microfluidic Fabrication

The PDMS sheets were made using soft lithography with a Sylgard 184 silicone elastomer kit (Dow Chemical Company, Midland, MI, USA), by mixing resin and curing agent in a 10:1 *w*/*w* ratio then placing in a desiccator with vacuum pump to remove air bubbles. The prepared mixture was poured into a simple flat rectangular mold to form a flat thin sheet, then cured in an oven at 70 °C for 45 min.

**Figure 2:**
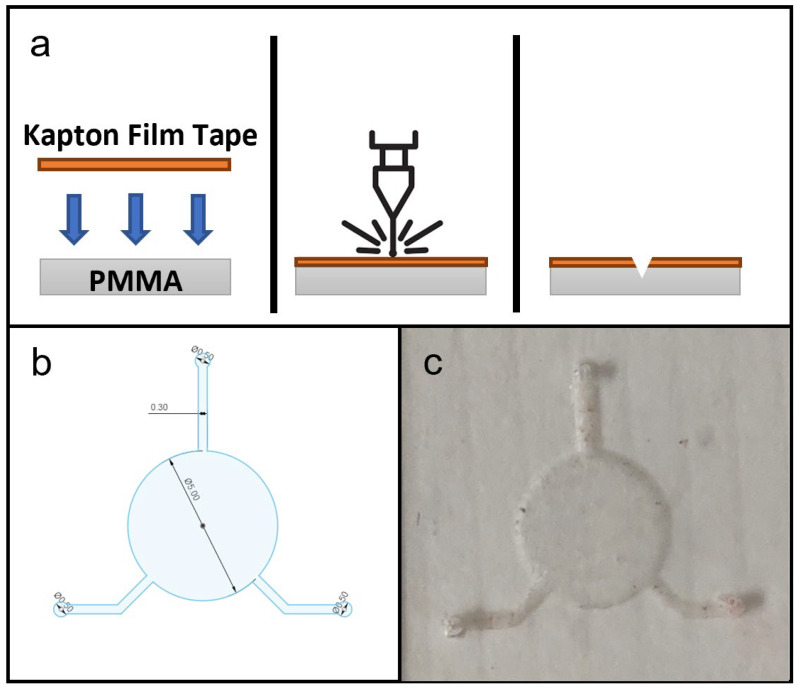
(**a**) Schematic diagram of fabrication microfluidic features on PDMS substrate, showing in cross-section the setup of materials, laser ablation process and microfluidic features on PDMS substrate. (**b**) CAD drawing of microfluidic design for this study. (**c**) Photograph of a produced PDMS microfluidic chip.

Figure 2a shows the general fabrication protocol for all the microfluidic components in this study. The laser ablation process was controlled by the software, Luban. The substrate materials used for testing were PDMS sheets. The substrates, with the Kapton film tape applied on top, were placed on the workspace, and laser ablation was performed at a constant speed of 140 mm/min and with laser power at 30%/480 mW, 40%/640 mW, 50%/800 mW, 60%/960 mW, 70%/1120 mW, 80%/1280 mW, 90%/1440 mW and 100%/1600 mW to create microchannels and other microfluidic features with different depths and widths. Afterwards, the remaining polyimide tape was removed and any carbon deposits from laser ablation were removed using IPA.

### 2.6. Microfluidic Dimension Test

Dimensional testing of laser-fabricated microfluidic features of PDMS was performed using straight microchannels of width 0.5 mm, with microchannel cross-sectional profiles measured for depth and width. For testing of flexible microfluidic chips for wearable devices, the design is shown in Figure 2b, which is based on a device for epidermal sweat analysis [3], with slight modifications. A Zeiss Microscope with an Axiocam 105 color digital microscope camera with Zeiss Zen 2.3 Lite software (Jena, Germany) was used to record digital images of microfluidic channel cross-section profiles and the top view for overall microfluidic chip designs.

### 2.7. Flexible Microfluidic Sensing Device Assembly

**Figure 3:**
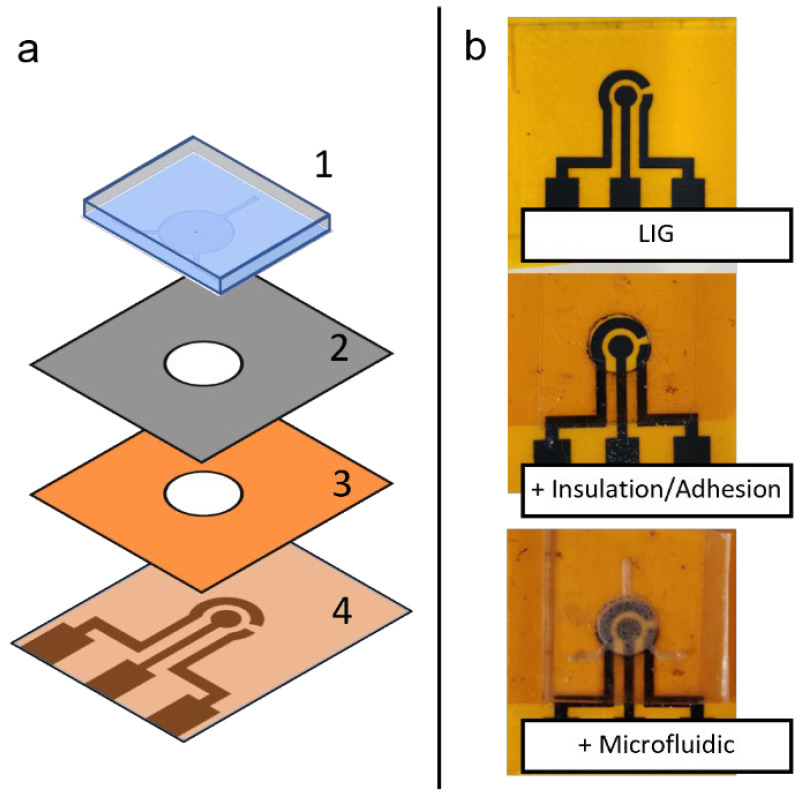
(**a**) Schematic depiction of assembly of a flexible microfluidic sensing device, showing the different layers of materials: (1) PDMS microfluidic chip, (2) adhesion layer and (3) insulation layer with circular cutout, and (4) LIG electrode unit. (**b**) Photographs of the assembly process, showing different layers being added on top of previous layers.

To assemble the flexible microfluidic sensing device, different components are arranged in layers in a specific order. From bottom to top, the layers are the laser-induced graphene electrode layer, insulation/adhesion layer and microfluidic layer. The insulation/adhesion layer consists of polyimide tape layer attached to 3M™ double-sided tape (Saint Paul, MN, USA) attached on top and laser-cut to create a circular opening for the electrochemical cell. During assembly, the three-electrode area of LIG, the circular opening in the insulation/adhesion layer and the circular chamber of the microfluidic chip are aligned by applying each layer manually with finger pressure. The assembly scheme is shown in Figure 3a.

### 2.8. Microfluidic Flow Testing

Microfluidic flow tests were performed to observe any leakage during normal flow operation. The test was first conducted on a flat surface and then on curved surfaces, to assess the suitability for flexible microfluidic operation. Two curved surfaces selected for testing were a safety goggle lens (Curve 1) and the side of a 500-mL laboratory glass bottle (Curve 2) to represent two levels of curvature. For all tests, a syringe pump (PHD Ultra Syringe Pump, Harvard Apparatus, Holliston, MA, USA) was used to provide a constant flow rate of 20 µL/min to simulate the collection and flow of perspiration on human skin into the microfluidic device.

## 3. Results and Discussion

### 3.1. LIG Fabrication

Figure 1c is a photograph of a fabricated LIG on Kapton film tape. In a comparison of the resulting LIG electrodes with the CAD drawing, the laser ablation protocol was shown to be capable of accurately reproducing the key design geometries of a three-electrode setup on a Kapton film tape surface.

The diode laser used in this study produced a beam of maximum power of 1600 mW at 450 nm. In most cases, CO_2_ laser machines are used in the fabrication of LIGs [11,16,18,22,24,25] and other microfluidic components on solid substrates [26,27,28]. Still, diode laser machines have also been used successfully to make LIGs [7,10,12] and microfluidic features on solid substrates [29]. While significantly less powerful in terms of laser output compared to CO_2_ laser machines, diode lasers are lower in cost, easier to operate and take up less space in the laboratory or workshop. Kapton film tape is considered an excellent choice as the key material for flexible microfluidic sensing devices formed by laser. Kapton film tapes have been commonly used in microfabrication and electronic facilities, including prototypes of printed circuit boards (PCB) as well as microfluidic chips and microelectrodes. Therefore, any of these facilities are highly likely to have Kapton film tapes available for immediate use. If not, they are easily obtained, due to their low cost and ready availability. The white printing paper was used as a placeholder for flexible substrates in this study. It can be replaced with more appropriate materials, such as medical-grade tapes or polymer sheets, to suit the requirements of the wearable device.

### 3.2. Optimization of LIG Fabrication

**Figure 4:**
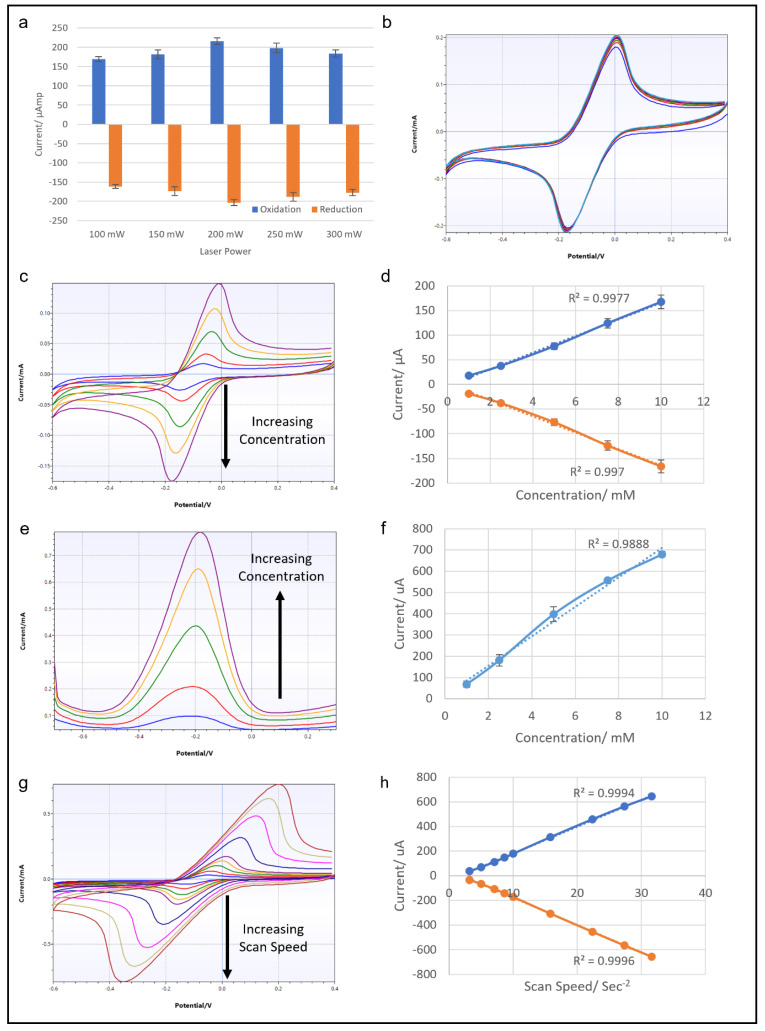
(**a**) Graph showing the results of CV from LIG electrodes fabricated by laser powers of 100, 150, 200, 250 and 300 mW at 100 mV/s scan rate. (**b**) Results of 10 cycles of CV with 10 mM ferricyanide with 100 mV/s scan rate from a LIG electrode made with a 200 mW laser. (**c**) CV traces of LIG electrode with ferricyanide concentrations of 1, 2.5, 5, 7.5 and 10 mM at 100 mV/s scan rate, and the corresponding graph relating the oxidation and reduction peak currents to ferricyanide concentration (**d**). (**e**) DPV traces of LIG electrode with ferricyanide concentrations of 1, 2.5, 5, 7.5 and 10 mM, and the corresponding graph showing the relation of the peak current to ferricyanide concentration (**f**). (**g**) CV traces from a LIG electrode with 10 mM ferricyanide at scan speeds of 10, 25, 50, 75, 100, 250, 500, 750 and 1000 mV/s, and the corresponding graph showing the relationship of oxidation and reduction peak currents to scan speed (**h**).

Multiple LIG electrode units were fabricated by use of different laser power levels, to determine the optimal settings for electrochemical sensing purposes. The laser power levels used were 100, 150, 200, 250 and 300 mW, with all other settings remaining the same. Then the electrochemical performance of each electrode was tested by cyclic voltammetry of 10 mM potassium ferricyanide solution. All the LIGs tested produced reversible CV traces with similar oxidation and reduction peak positions and amplitudes. However, the peak measurements indicated that LIG from the 200 mW laser setting produced the highest and lowest oxidation and reduction peaks (Figure 4a). Therefore, a laser power of 200 mW was selected for LIG fabrication for the rest of the study.

### 3.3. Electrochemical Characterization

Another set of CV tests was conducted with a LIG electrode fabricated with a diode laser output of 200 mW. First, 10 mM potassium ferricyanide solution was used to run 10 consecutive cycles of CV. The results shown in Figure 4b proved that the LIG surface remained stable after repeated CV cycles, with no noticeable deterioration of the electrochemical performance. To test the concentration dependence of the response, potassium ferricyanide solutions with concentrations of 1–10 mM were tested in a series of CVs. The results in Figure 4c,d show CV traces for potassium ferricyanide solution of 1, 2.5, 5, 7.5 and 10 mM and the accompanying graphs of oxidation and reduction peaks. The CV traces showed both oxidation and reduction peaks expanding as the concentration of test analyte solution increased, as expected. The plotted data show R^2^ values over 0.99 for both oxidation and reduction current peaks, indicating a linear correlation with all concentrations of the samples. A similar concentration test was performed with differential pulse voltammetry (Figure 4e,f). However, the linear correlation of DPV oxidation peaks was not as strong as in the CV results, yielding an R^2^ value of around 0.988. The last CV test explored the effect of scan speed on LIG electrode. Figure 4g,h show the results of CV at scan speeds of 10, 25, 50, 75, 100, 250, 500, 750 and 1000 mV/s, with the corresponding data plotted on a graph. The CV traces clearly show increasing current response as the scan speed increased, and this relationship was highly linear, with an R^2^ value of over 0.99. Overall, this series of electrochemical tests prove the LIG electrodes to be stable and reliable conductive material, suitable for use in electrochemical sensing.

It is important to note that, for optimal and consistent results, the best practice would be to select specific Kapton tape with a known brand and model that can be readily restocked when a new roll of tape is needed. This is due to slight differences in the Kapton tape properties between various manufacturers and models that result in LIG with a small variance in electrochemical performance, as seen in changes in CV peaks amplitudes.

### 3.4. Raman Spectroscopy for LIG Characterization

**Figure 5:**
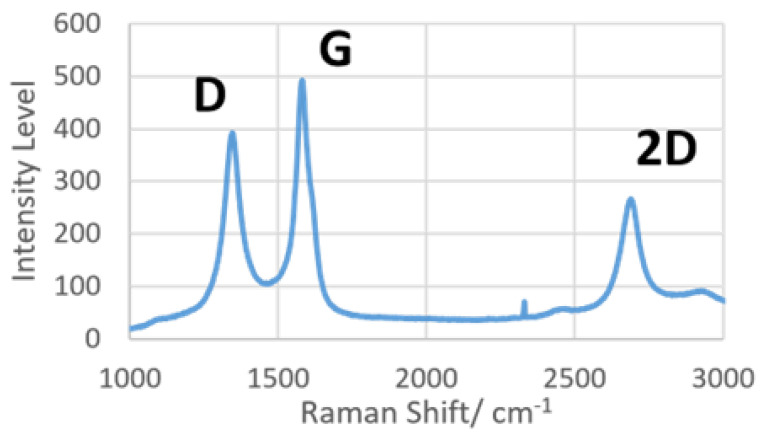
Raman spectra of laser-induced graphene produced with 200 mW laser power.

The LIG was subjected to dispersive Raman spectroscopy to prove the presence of graphene on the surface. Focusing on the spectrum between 1000 and 3000 cm^−1^, there are three distinct peaks of interest, namely, D, G and 2D. Briefly, peak D (~1350 cm^−1^) indicates the level of defects in *sp^2^*-hybridized carbon structures, peak G (~1580 cm^−1^) shows the presence of graphene, and peak 2D (~2700 cm^−1^) was caused by second-order resonance, correlated to the number of graphene layers present [10]. The results from Raman spectroscopy are shown in Figure 5, with all three characteristic peaks of LIG present in the spectrum. A previous Raman spectroscopy study [30] had determined that the ratio of integrated intensities of peaks D and G (*ID*/*IG*) is inversely proportional to the size of crystalline nanographite in the materials. Referring to Figure 5, the intensity of peak G was clearly higher than that of peak D, indicating the formation of a crystalline graphitic layer on LIG. The result of Raman spectroscopy conclusively proved that the laser ablation protocol on polyimide material used in this study could effectively create graphene.

### 3.5. Microfluidic Fabrication

The material chosen for flexible microfluidic chip construction was PDMS, as the elastomer has been widely used as the standard material for other microfluidic fabrication methods, including photolithography and soft lithography. Therefore, any facilities that engage in microfluidic research or fabrication will be likely to have PDMS kits in their inventory, and appropriate technical experience of how to handle the material. As with Kapton film tape, the availability of a PDMS kit should allow fabrication of flexible microfluidics to be carried out immediately.

### 3.6. Microfluidic Dimension Measurement

**Figure 6:**
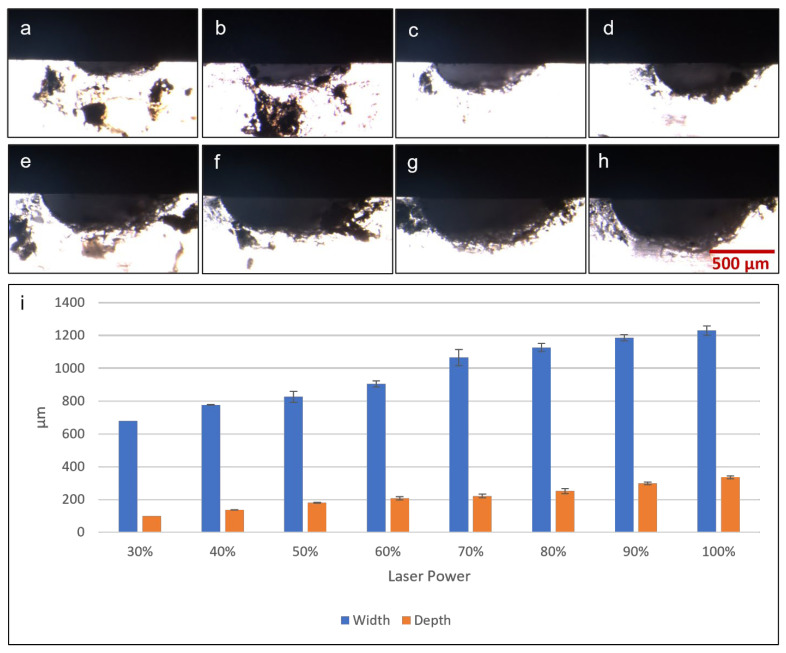
Microscopic photographs of microchannel cross-sections fabricated by diode laser with the following laser power: (**a**) 30%/480 mW, (**b**) 40%/640 mW, (**c**) 50%/800 mW, (**d**) 60%/960 mW, (**e**) 70%/1120 mW, (**f**) 80%/1280 mW, (**g**) 90%/1440 mW and (**h**) 100%/1600 mW. (**i**) Graph showing results of microchannel cross-section dimensions (width and depth) measured from microscopic photographs.

The first microfluidic fabrication test was to determine the cross-sectional profile and dimensions of microchannels fabricated by laser ablation at different laser powers. A microchannel of 0.5 mm width was used as the test model and was fabricated with laser power set at 320, 480, 640, 800, 960, 1120, 1280, 1440 and 1600 mW. These values corresponded to in-software laser settings of 20% to 100%, in 10% increments. Figure 6a–h shows microscopic photographs of cross-sections of fabricated microchannels, the white area being PDMS against a black background, clearly showing a semi-elliptical cross-sectional profile with an elongated horizontal axis. Figure 6i shows graphically the depths and widths of microchannels fabricated at different laser powers. A clear trend was observed, in which higher laser power used in fabrication resulted in wider and deeper microchannel dimensions. At the lowest power tested (320 mW), there was no visible microchannel on the PDMS surface, and thus no recorded data on the graph. The lowest setting to produce a visible microchannel was 480 mW, which resulted in a depth of around 0.1 mm and a width of around 0.68 mm, wider than the design width of 0.5 mm. Increasing the laser power for fabrication resulted in microchannel widths that were even wider than the target of 0.5 mm, up to 1.2 mm at the highest laser setting. Microchannel depth also increased as greater laser power was applied, resulting in depths of around 0.1 mm to 0.35 mm across the range of power output tested. Overall, this laser fabrication protocol can create basic microfluidic features including microchannels of varying dimensions, depending on the laser output. While the results showed that the microchannel dimensions fabricated did not perfectly match the width specified in CAD drawings, simple adjustments and optimizations can be quickly performed, to yield satisfactory outcomes.

### 3.7. Flexible Microfluidic Sensing Device Assembly

The CAD drawing of the microfluidic design used for the flexible microfluidic sensing device and an actual photograph of the laser-ablated microfluidic chip are shown in Figure 2c. The design consisted of one 5-millimetre-diameter circular chamber to accommodate the electroactive area of LIG electrodes, and three microchannels of 0.3 mm width leading to the central chamber, which served for fluid collection and delivery. In its original configuration [3], the device would be attached to the subject’s skin to collect a sweat sample and send it to the chamber for detection. For this study the two microchannels were connected to a syringe pump that delivered sample fluids to the chamber while the remaining channel acted as an outlet. A hole puncher (0.5 mm in diameter) was used to create holes for inlets and outlets for fluid flow. Silicone tubes with a 0.8 mm inner diameter were used for infusion. Based on the effects of laser power levels on microchannel dimension during laser fabrication, the microfluidic chip was laser-ablated at 800 mW power output to create a microchannel depth of approximately 0.18 mm. This particular power setting was selected because microchannels produced with lower laser power settings did not have sufficient depth for uninterrupted, continuous fluid flow.

**Figure 7:**
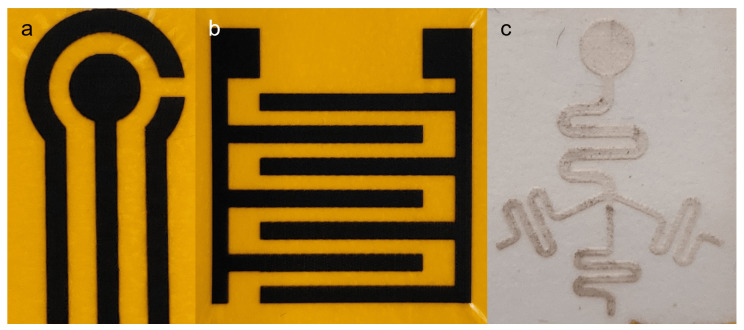
Photographs showing additional designs fabricated by the protocol presented. (**a**) LIG designed after commercial screen-printed electrode (SPE). (**b**) Interdigitated LIG electrode. (**c**) Microfluidic test design combining different elements, including straight and curved lines of different widths, intersection with branching multiple channels, and circular geometry in a single unit.

Different components fabricated in this study (LIG and microfluidic chip) can be assembled to form a flexible microfluidic sensing device. Additional examples of LIGs and the microfluidic chip fabricated using this protocol are shown in Figure 7. Another required component was an insulation/adhesion layer between the LIG and microfluidic parts. This layer consisted of Kapton film tape at the bottom with 3M double-sided tape applied on top, with a laser-cut circular opening for the LIG electrodes and sample fluid chamber. The Kapton film tape side was applied on top of the LIG electrode to serve as insulation, restricting fluid contact to the active electrode area in the circular opening. The double-sided tape functioned as an adhesive joining the microfluidic chip to the LIG layer. Layer-by-layer photographs of the assembly process are shown in Figure 3b.

### 3.8. Microfluidic Flow Test

**Figure 8:**
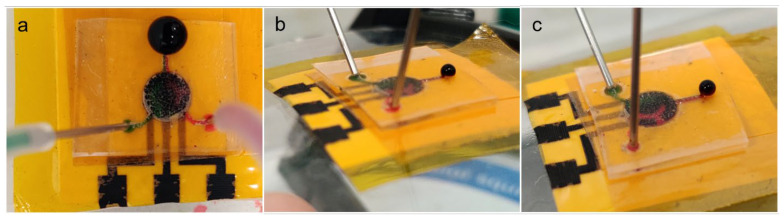
Photographs showing microfluidic flow testing of an assembled flexible microfluidic sensing device with a flow speed of 20 µL/min under different surfaces: (**a**) flat surface, (**b**) curved surface (safety goggle lens) and (**c**) body of a 500-mL laboratory glass bottle.

The assembled flexible microfluidic sensing device was subjected to microfluidic flow using a syringe pump with the flow rate set at 20 µL/min, to simulate body sweat entering the microchannels and collecting inside the main chamber. In the test, red and green dyes were infused into either side of the main chamber through the left or right inlet microchannels. The flow test was performed while the device was lying flat or attached to two surfaces with different curvatures. Figure 8 shows photographs of the flow tests on all three surfaces, all of which produced the same result. The photographs show green dye entering via the left microchannel and red dye entering via the right microchannel. The two solutions accumulated in the main chamber with minimal mixing inside, as observed by two distinct halves of different colors. Distinct dye separation was also observed as fluid flow exited the main chamber, entering the outlet microchannel with two clear colored segments. Also, there was no observable fluid leakage along the path of the microfluidic device. The devices on curved surfaces were shown to adhere tightly to the test surfaces and to maintain structural integrity with no visible defects, such as layers peeling off. This set of tests proved that this fabrication protocol can efficiently produce a functional flexible microfluidic sensing device that functions as a test bed for wearable devices for continuous healthcare monitoring on human subjects.

### 3.9. Microfluidic CV Test

**Figure 9:**
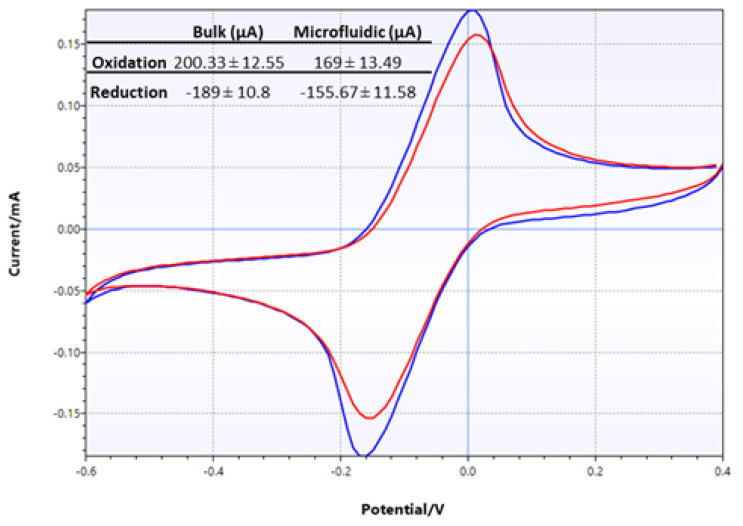
CV traces of LIG, comparing test results from bulk setting (blue) and microfluidic setting (red) performed with 10 mM potassium ferricyanide. The numerical data are shown in the inset table.

Electrochemical characterization of LIG electrodes was comprehensively conducted as described above. However, it was deemed necessary to perform another test when the LIG electrode was assembled into a microfluidic device. CV tests were performed in two different configurations: in bulk solution, where the test analyte was dropped directly onto electrodes, and in microfluidic configuration, where the test analyte was infused into the assembled flexible microfluidic sensing device. The results of two sets of CV tests using 10 mM potassium ferricyanide are shown in Figure 9. The CV traces clearly showed that the two different test conditions produced very similar results in terms of peak height and positions. However, closer examination of the oxidation and reduction peaks from these two sets of data showed that the current produced from the microfluidic set was lower than that from the bulk solution test. Specifically, oxidation peak currents were 200 µA and 169 µA in bulk solution and microfluidic configuration, respectively, while reduction peak currents were −189 µA and −156 µA.

## 4. Conclusions

This study has shown that simple combinations of a diode laser engraving machine and Kapton film tape can be used to effectively fabricate both flexible microfluidic chips and laser-induced graphene sensing elements, which are combined to form the basis of a functional wearable device. Both LIG electrodes and microfluidic chips can be built independently, allowing multiple combinations of electrode configuration and microfluidic design to suit the required experimental parameters and objectives. The fabrication protocol proposed here represents, arguably, the most economical and the least technically demanding method of creating PDMS-based, flexible microfluidic sensing devices that can be performed completely in-house. A layperson can use hobbyist-level equipment with bundled software and materials to produce basic, functioning prototypes on a lab benchtop. With only a single instrument and a handful of materials needed for both fabrication of LIG electrodes and flexible microfluidic chips, the entire process is highly streamlined and simplified, enabling completion of the final product in under 1 h. With an estimated start-up cost of approximately USD 1000 and an estimated production cost of around USD 1 per unit, this fabrication method presents itself as a highly cost-effective option. In contrast, conventional photolithography for microfluidic fabrication that demands highly advanced facilities operated by trained personnel would require a much longer time for fabrication of microfluidic component alone, after which another system would be required to create graphene electrodes. One viable option is a CO_2_ laser machine, which costs more, occupies a larger space in the lab, and is much more complex to operate. These two separate processes must be performed at different facilities using their own sets of protocols and materials before assembly and testing are possible. The fabrication method presented here eliminates most of these issues.

Ultimately, the goal is to advance the development of microfluidics systems to a point where designing and producing custom-made microfluidic devices for use as either replacements for standard laboratory equipment or prototyping of lab-on-a-chip or point-of-care diagnostic devices is feasible, practical and productive to research end objectives.

## Data Availability

All data are included in the manuscript.
